# Clinical characteristics of *Treponema denticola*-associated lung abscess diagnosed by metagenomic next-generation sequencing: a case series analysis

**DOI:** 10.3389/fcimb.2025.1688498

**Published:** 2025-11-11

**Authors:** Hangdi Xu, Yueqing Xu, Jing Gu, Xiaoling Wang, Bo Liao, Pengpeng Zhou, Shengjun Wu, Ran Tao, Ying Fu

**Affiliations:** 1 Department of Pulmonary and Critical Care Medicine, Regional Medical Center for National Institute of Respiratory Disease, Sir Run Run Shaw Hospital, School of Medicine, Zhejiang University, Hangzhou, Zhejiang, China; 2 Department of Pulmonary and Critical Care Medicine, the First Division Hospital of Xinjiang Production and Construction Corps, Akesu, China; 3 Guangzhou University of Chinese Medicine, Guangzhou, China; 4 Department of Hyperbaric Oxygen Medicine and Rehabilitation, General Hospital of Southern Theater Command of People's Liberation Army (PLA), Guangzhou, China; 5 Department of Clinical Laboratory, Sir Run Run Shaw Hospital, School of Medicine, Zhejiang University, Hangzhou, Zhejiang, China; 6 Zhejiang Provincial Engineering Research Center of Innovative Instruments for Precise Pathogen Detection, Zhejiang University, Hangzhou, Zhejiang, China; 7 Department of Pathology, Sir Run Run Shaw Hospital, Zhejiang University School of Medicine, Hangzhou, China

**Keywords:** Treponema denticola, lung abscess, clinical characteristics, metagenomic next-generation sequencing, case series

## Abstract

**Introduction:**

*Treponema denticola* is an oral anaerobic bacterium commonly associated with periodontitis, but its role in lower respiratory tract infections (e.g., lung abscess) has long been overlooked. For bacteria that grow anaerobically, traditional culture methods exhibit low detection rates, which directly lead to the mis-diagnosis of anaerobic infection. With the ultilization of metagenomic next-generation sequencing (mNGS) in clinical practice, we studied the clinical features and treatment strategies of *T. denticola*-associated lung abscess.

**Methods:**

A retrospective analysis was conducted on patients confirmed with *T. denticola* lung abscess by mNGS from October 2023 to October 2024. Routine aerobic bacterial culture and stains were used. Histopathological analysis and Warthin-Starry silver staining was completed on samples from lung tissue. A literature review was performed using PubMed and CNKI (up to June 2025).

**Results:**

Seven patients were diagnosed with *T. denticola* lung abscess under mNGS. The cohort predominantly comprised elderly males (mean age 62.3 years), all of whom had underlying oral diseases. Clinical manifestations featured chronic cough (mean symptom duration 3.6 months) and frequent hemoptysis (85.7%), with notably mild systemic inflammation (only one febrile case). Characteristic CT findings included mass-like lesions with necrosis (100%) and cavitation (71.4%), without air-fluid levels. Conventional cultures were overwhelmingly negative, whereas mNGS detected *T. denticola* in all seven cases. Among the seven patients, one showed clinical improvement after two months of amoxicillin-clavulanate therapy, and another responded well to seven months of doxycycline treatment. The remaining five patients initially treated with levofloxacin or moxifloxacin demonstrated poor responses, with three cases ultimately requiring surgical resection of the lesions.

**Discussion:**

*T. denticola* lung abscess is most commonly seen in individuals with poor oral hygiene. It presents as an indolent, chronic course and a high incidence of hemoptysis. Typical CT findings include a mass-like lesion with cavitation but no air–fluid level. Traditional microbiological detection often yield false negative results, making mNGS a critical diagnostic tool. First-line therapy should include β-lactams or tetracyclines, and surgery is warranted for refractory cases or massive hemoptysis.

## 1 Introduction

*Treponema denticola* (*T. denticola*) is a Gram-negative, obligate-anaerobic spirochete that is widely distributed in human subgingival plaque (Ishihara, 2010; Goetting-Minesky et al., 2021). It is classically associated with periodontitis and can form intricate biofilms with other oral anaerobes—such as *Porphyromonas gingivalis* and *Tannerella forsythia*—thereby synergistically amplifying pathogenicity and promoting the development of periodontal abscesses (Dashper et al., 2011; Ng et al., 2019). When host immunity is compromised or the oral microbiota becomes dysbiotic, oropharyngeal organisms can be aspirated or disseminated hematogenously to the lower respiratory tract, resulting in pulmonary infection and abscess formation (Bartlett, 2005). Although the role of *T. denticola* in periodontitis is well established, its contribution to lung infections has long been overlooked. This neglect stems largely from the fastidious culture requirements of anaerobes-conventional culture methods demonstrate remarkably low detection rates for anaerobic spirochetes. Studies report positive culture rates below 30% for sputum or pus specimens, with some series reporting 0% in positive rate (Su et al., 2022; Zhang et al., 2025).

Metagenomic next-generation sequencing (mNGS) has revolutionized the diagnosis significantly, especially in anaerobic infections. Clinical data demonstrate that mNGS achieves 67.7% positive rate in etiological diagnosis of lung abscesses, markedly higher than that of traditional culture (Su et al., 2022; Zhang et al., 2025). Notably, mNGS boosts anaerobic pathogen detection to 93.8%-100%, with particular diagnostic advantages for sterile-site specimens like biopsy tissues or pleural fluid (Chen et al., 2021b).

In contrast to the absence of reported cases in our hospital over the previous five years, we report seven cases of *T. denticola* -associated lung abscesses diagnosed during the first year following the introduction of mNGS. We further analyzed their clinical manifestations, diagnostic approaches, and treatment strategies. In addition, we conducted a comprehensive review and in-depth analysis of previously reported cases through a systematic literature search. Our findings not only expand the current understanding of *T. denticola*-associated pulmonary abscesses but also provide evidence to support the optimization of management strategies for refractory cases.

## 2 Patients and methods

### 2.1 Patients

This retrospective study screened patients with lung abscesses treated at Sir Run Run Shaw Hospital, School of Medicine, Zhejiang University from October 2023 to October 2024. mNGS of bronchoalveolar lavage fluid (BALF) or lung biopsy specimens were collected for pathogenic identification, consistent with clinical evidence. The association between lung abscess and *T. denticola* was confirmed by two senior pulmonologists and one senior laboratory physician. The study ultimately enrolled seven patients with *T. denticola*-associated lung abscesses. Comprehensive clinical data were collected, including demographic characteristics (age, sex), underlying diseases, clinical manifestations, disease progression, laboratory and radiological findings, molecular diagnostic results, treatment regimens, and prognosis.

### 2.2 Assessment of oral health status

All patients underwent a clinical oral examination performed by a dentist, including assessments for dental caries, periodontal status, dental plaque, and dental calculus. The diagnosis of periodontitis was based on the criteria established at the 2017 World Workshop on the Classification of Periodontal and Peri-Implant Diseases and Conditions (Papapanou et al., 2018). Dental calculus and plaque were evaluated according to the Chinese Stomatological Association’s guideline, “Standard of periodontal examination and evaluation during oral diagnosis and treatment” (Society of Periodontology, 2021). Poor oral hygiene was defined as a Plaque Index score ≥3 (Society of Periodontology, 2021; Lertpimonchai et al., 2017) or a Calculus Index score ≥2, which indicates the presence of subgingival calculus.

### 2.3 Traditional microbiological methods

Different processing procedures were applied to BALF and lung tissue samples. For BALF samples, centrifugation at 3500 rpm was performed prior to culture and staining, and the resulting pellet was used for subsequent analyses. Lung tissue samples were used directly for further detection. Three types of culture media (Bioivd Biotechnology [Zhengzhou] Co., Ltd.) were used for bacterial isolation and identification, including Columbia Blood Agar, Haemophilus influenzae Agar (HAE), and Sabouraud Dextrose Agar (SDA). All plates were incubated under 5% CO^2^ at 35 ± 2°C for up to seven days. Suspected colonies growing on the plates were subsequently subcultured and subjected to species-level identification using Matrix-Assisted Laser Desorption/Ionization Time-of-Flight Mass Spectrometry (MALDI-TOF MS; bioMérieux Shanghai Biotech Co., Ltd.). In addition, Gram staining, fungal fluorescence staining, and acid-fast staining (Wuhan Baso Medical Device Co., Ltd.) were performed for the detection of bacteria, fungi, and *Mycobacterium* spp., respectively. Anaerobic culture was not involved. The identification and interpretation of suspected colonies isolated from BALF were performed according to the IDSA/ATS guidelines (Kalil et al., 2016). The interpretation of pathogens and normal flora are based on references from The ABX Guide and Manual of Clinical Microbiology (11th Edition) (University., 2024; David et al., 2015).

### 2.4 mNGS test

BALF or lung tissue samples were collected and then transported on dry ice to clinical laboratory within 4 hours for mNGS testing. Following the established protocol, cellular materials in samples were lysed and enriched. Total nucleic acid extraction kits and mNGS DNA library preparation kits (Tiangen Biotech Co., Ltd, China) were used for sequencing library construction. Metagenomic sequencing was performed on the BGISEQ platform, and bioinformatic analysis was conducted following the standardized mNGS protocol established by Dian Diagnostics Group Co., Ltd (Hangzhou, China) (Sun et al., 2024), with additional species-specific validation steps for *T. denticola.* Briefly, the raw sequencing data were processed through quality control using fastp (v0.24.1) to remove adapter sequences and filter out low-quality reads. Host-derived sequences were subsequently removed by alignment to human reference genomes (hg38, YH genome, and T2T-CHM13) using BWA-MEM (v0.7.17-r1188). Non-human reads were taxonomically classified by alignment against an in-house pathogenic microorganism database using BWA-MEM. For *T. denticola* confirmation, candidate reads were aligned to the reference genome of the *T. denticola* type strain (GenBank accession: NC_002967.9) using BWA-MEM. Read counts were quantified using custom in-house scripts. To ensure species specificity, pathogen-specific reads were further validated by BLASTn against the NCBI nucleotide (nt) database. Detection reliability was ensured through the inclusion of negative control. Metagenomic libraries for each sample yielded ≥ 20 million high-quality reads, with a mean Q30 score ≥ 90%. The relative abundance of *T. denticola* was first calculated as the proportion of its mapped reads among the total microbial reads. No *T. denticola* signal was detected in the seven negative controls processed within the same batch.

### 2.5 Histopathological analysis

Lung tissue specimens were fixed in 10% neutral buffered formalin for at least 24 hours. Following fixation, the tissues were dehydrated through a graded ethanol series, cleared in xylene, and embedded in paraffin. Sections were cut at a thickness of 4-5 μm using a microtome and subsequently stained with hematoxylin and eosin (H&E) according to standard protocols (Dunn et al., 2024). For the histopathological detection of spirochetes, Warthin-Starry staining was conducted following an established laboratory protocol based on the method of Graham et al (Graham et al., 2018).

### 2.6 Literature review

To comprehensively identify all published studies on lung abscess associated with *T. denticola*, two investigators (Hangdi Xu and Ying Fu) independently searched PubMed and China National Knowledge Infrastructure (CNKI) up to June 30, 2025. The search terms were the following key words: (“odontogenic flora” or “*Treponema denticola*” or “*T. denticola*” or “red-complex”) and (“lung abscess” or “lung infection” or “pneumonia”).

## 3 Results

### 3.1 General clinical features

The basic characteristics of the seven patients in our cohort are summarized in **Table 1**. The cohort predominantly consisted of elderly males (age range: 47–77 years), with five patients aged over 60 years. None of the patients had documented immunodeficiencies or histories of immunosuppressive therapy. Three patients had underlying comorbidities, including diabetes mellitus, coronary heart disease, and hypertension. All patients exhibited poor oral hygiene (as defined in the methods) with significant dental plaque (mean index: 3.57) and calculus accumulation (mean index: 1.71) (**Supplementary Table 1**). Patients with *T. denticola*-associated lung abscess typically present with a chronic disease course, demonstrating an average diagnostic delay of 3.6 months from symptom onset to definitive diagnosis. The clinical manifestations are mainly respiratory symptoms such as cough and sputum production, while systemic inflammatory responses are relatively mild, with only one case accompanied by fever. It is worth noting that over 85% of patients (6/7 cases) experienced hemoptysis.

**Table 1:** Clinical information of patients with *Treponema denticola* lung abscess.

Case	Age	Sex	Underlying disease	Smoking/ drinking	Poor oral hygiene	Symptoms	Time	WBC 10^9^/L	NEU 10^9^/L	Hgb g/L	PCT ng/mL	CRP mg/L	Lesion site	Lesion size (mm)
1	69	male	HPT	Y/Y	Y	Cough and sputum, hemoptysis	5M	5.8	4.08	97	–	51.6	Right upper lung mass with necrosis and cavity	55*54
2	60	male	–	Y/N	Y	Fever, cough and sputum, hemoptysis	2M	10.7	9.63	105	<0.04	59	Left lower lung mass with necrosis and cavity	51*48
3	55	male	DM	N/N	Y	Cough and sputum, hemoptysis	3M	10	8.98	139	<0.04	1.1	Right lower lung mass with necrosis and cavity	21*14
4	61	female	–	N/N	Y	Cough and chest pain	3M	5.7	3.42	127	<0.04	10.2	Right upper lung mass with necrosis and cavity	45*26
5	77	male	–	N/N	Y	Cough and hemoptysis	10M	5.1	2.43	122	–	6	Right upper lung mass with cavity	43*23
6	47	male	–	Y/N	Y	Cough and sputum, hemoptysis	1.5M	10.6	7.85	147	–	1.6	lingular segment mass with necrosis	28*22
7	67	male	DM, HPT	N/N	Y	Hemoptysis, dyspnea	1M	11.9	9.67	147	<0.04	125	Right lower lung mass with necrosis and cavity	67*54

M, months; DM, diabetes; HPT, hypertension; N, no; Y, yes; WBC, white blood cell; NEU, neutrophil; Hgb, hemoglobin; PCT, procalcitonin; CRP, C-reactive protein.

### 3.2 Laboratory tests, chest CT and bronchoscopy

The results of routine blood tests upon admission showed that 4 patients had a mild increase in white blood cell count, while the remaining 3 patients had normal results, with a median of 8.5 × 10 ^9^/L (5.1-10.6 × 10 ^9^/L). CRP levels were normal or mildly elevated (<60 mg/L) in 6 patients, with only one case exceeding 100 mg/L (36.2 mg/L, 1.1–125 mg/L). The procalcitonin (PCT) levels of the patients are normal.

**Figure 1:**
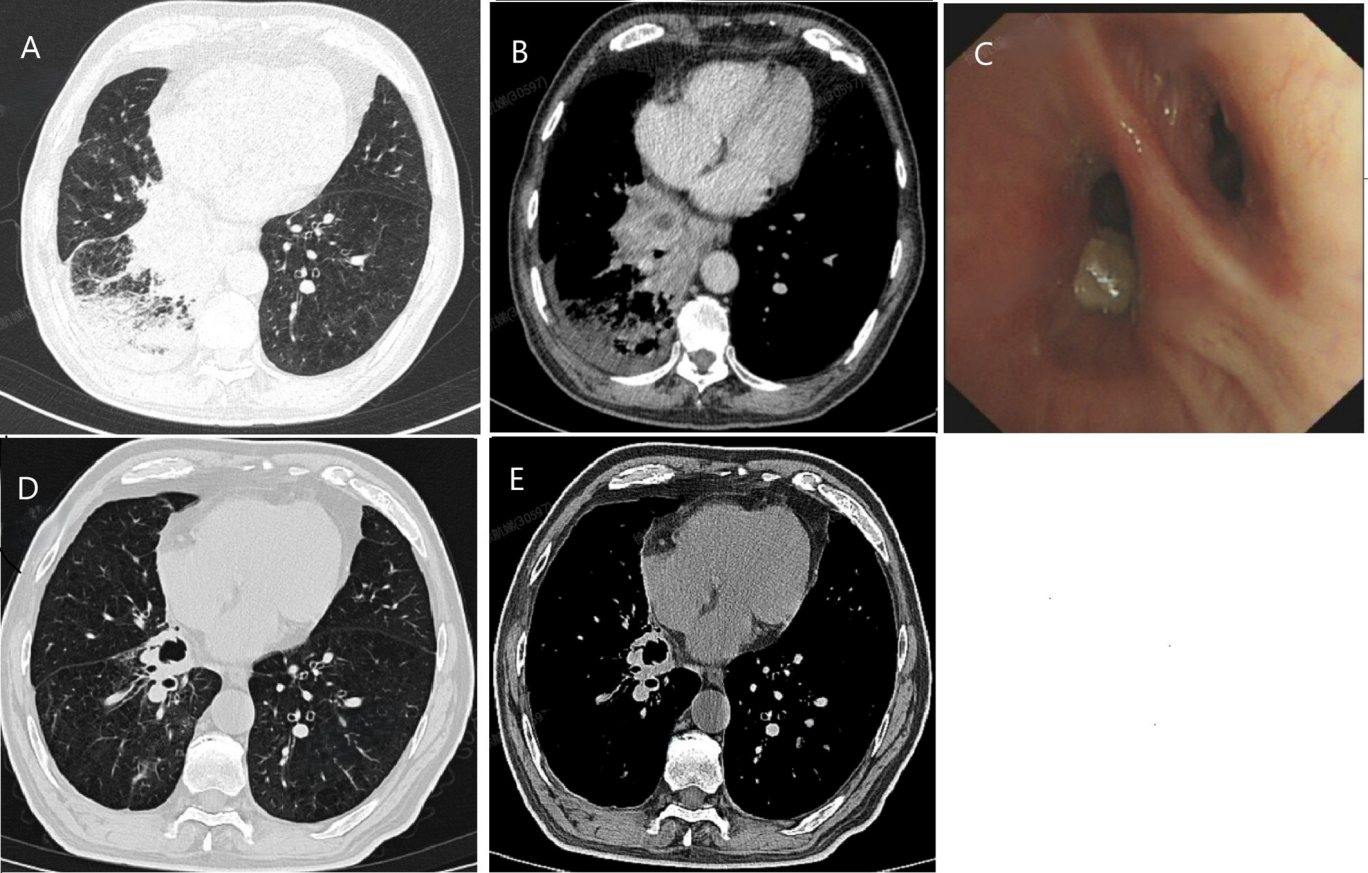
The initial chest CT of case 7 demonstrated mass-like opacities and necrosis (**A**, lung window; **B**, soft tissue window). After 6 months of treatment, a follow-up lung CT scan showed significant absorption and improvement of the lung lesion, with residual cavities (**D**, lung window; **E**, soft tissue window). Bronchoscopy examination shows purulent discharge in the lumen of the basal segment branch of the right lower lobe **(C)**.

**Figure 2:**
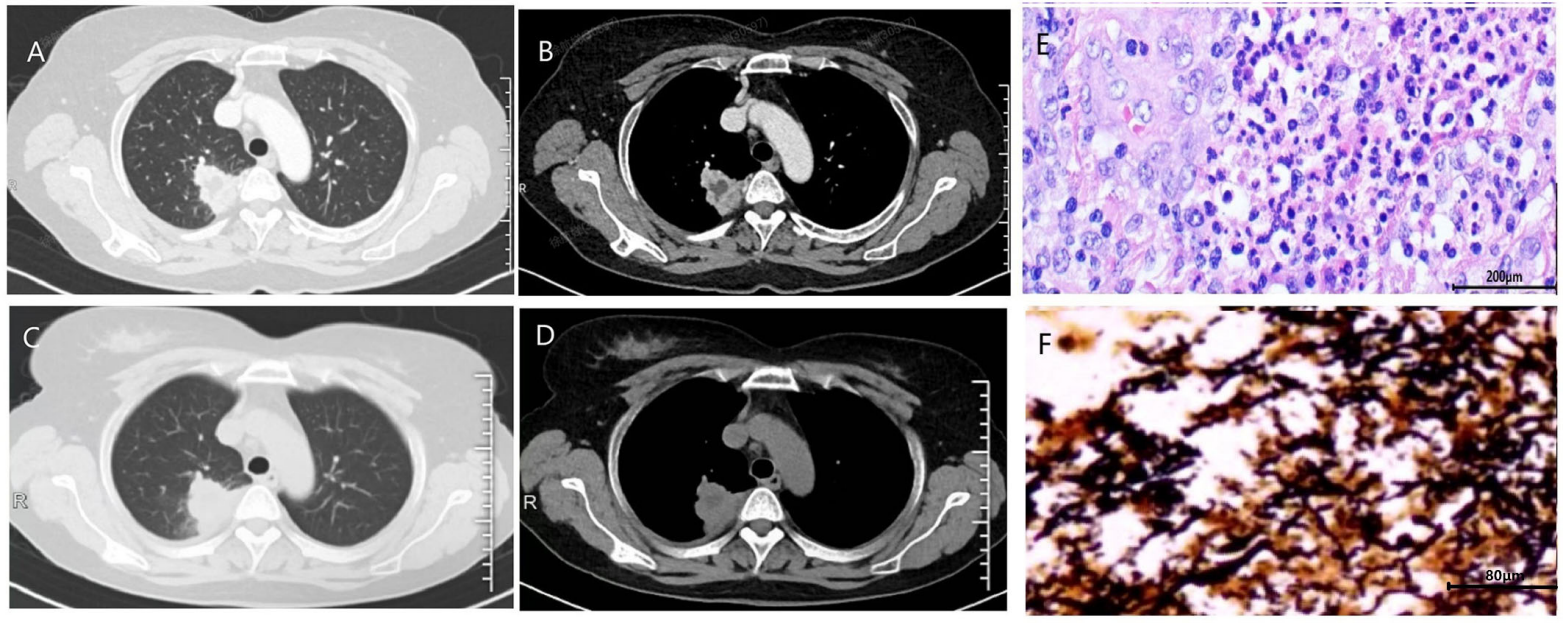
The initial chest CT of case 4 demonstrated mass-like opacities and necrosis (**A**, lung window; **B**, soft tissue window). After 1 month of treatment with levofloxacin and 10 days of treatment with cefmetazole, the patient’s lung lesions enlarged (**C**, lung window; **D**, soft tissue window). Histopathological examination of the surgically resected lesions revealed characteristic features of chronic lung abscess **(E)**. Warthin-Starry silver staining revealed numerous black, spiral-shaped bacteria, confirming the presence of spirochetes **(F)**.

Chest CT demonstrated mass-like opacities with necrosis in all seven cases, among which five exhibited cavitation without air-fluid levels (**Figures 1**, **2**). The lesions were distributed in Right upper lobe (3 cases), Right lower lobe (2 cases), Left lingular lobe (1 case), and Left lower lobe (1 case). The bronchoscopic manifestations included congestion and edema of the affected bronchial mucosa with purulent discharge within partial bronchial lumina (**Figure 1**).

### 3.3 Microbiological examinations

The microbiological etiology of the seven patients is summarized in **Table 2**. mNGS performed on four BALF or three lung tissue samples revealed a spectrum of oral anaerobic pathogens. Bacteria belonging to the *Treponema* genus were identified in all seven cases. Furthermore, in six cases, the *Treponema* genus constituted one of the three most abundant microbial groups in the community. A critical finding was that *T. DeNicola* represented a substantial proportion (ranging from 22.2% to 60.9%) of the total *Treponema* population in the cases where this specific quantification was available, underscoring its dominance within this genus (**Supplementary Table 2**). In contrast, conventional pathogen cultures of BALF or sputum were overwhelmingly negative, with *Streptococcus constellatus* isolated in only a single case (**Table 2**).

**Table 2:** Diagnosis, treatment, and prognosis of patients with *Treponema denticola* lung abscess.

Case	Samples for mNGS test	mNGS Results				Bacterial culture results	WS	Medication treatment	Surgical intervention	Prognosis
		Pathogens (genus)	Relative abundance (%)	Specific metagenomic reads	*T. denticola* Ratio^#^					
1	BALF	*Porphyromonas*	44.48	135174	/	NF	+	Levofloxacin 0.5g once a day for 10 days, Sitafloxacin 50mg every 12 hours for 3months.	Yes	Cured with resection
		*Streptococcus*	12.70	33514	/					
		*Treponema*	7.53	45309	22.2%					
		*Selenomonas*	1.25	4541	/					
		Prevotella*	31.98	176368	/					
2	Lung tissue	*Parvimonas*	22.53	83	/	ND	–	Levofloxacin 0.5g once a day for 1 months	Yes	Cured with resection
		*Treponema*	14.29	91	42.9%					
		*Tannerella*	10.44	78	/					
		*Peptostreptococcus*	7.69	33	/					
		*Fusobacterium*	6.59	36	/					
3	BALF	*Treponema*	83.37	10433	52.0%	NF	/	Moxifloxacin 0.4g once a day for 2 months	No	Co-infected with NTM, infection persists
		*Tannerella*	6.48	889	/					
		*Porphyromonas*	2.45	283	/					
4	Lung tissue	*Tannerella*	54.59	2153430	/	ND	+	Levofloxacin 0.5g for 1 month, Cefmetazole 1g every 8 hours for 10 days.	Yes	Cured with resection
		*Porphyromonas*	26.78	897923	/					
		Treponema*	15.65	555456	26.3%					
		*Campylobacter*	1.65	75130	/					
5	Lung tissue	*Treponema*	87.72	6105122	60.9%	*S. constellatus*	+	Doxycycline 0.1g every 12 hours for 7 months	No	Cured with medicines
		*Campylobacter*	4.11	549318	/					
		*Streptococcus*	3.09	196958	/					
		*Parvimonas*	2.56	150297	/					
6	BALF	*Treponema*	18.68	139200	44.8%	NF	/	Amoxicillin clavulanate 375mg every 8 hours for 2 months	No	Cured with medicines
		*Campylobacter*	1.54	7509	/					
		Rothia *	1.83	8454	/					
		Prevotella *	66.52	469101	/					
7	BALF	*Treponema*	54.78	182982	47.0%	NF	/	Moxifloxacin 0.4g once a day for 2 months, Sitafloxacin 50mg every 12 hours for 6 months	No	Cured with medicines
		*Campylobacter*	7.50	26533	/					
		*Porphyromonas*	6.38	16934	/					
		*Streptococcus*	4.21	5908	/					

BALF, bronchoalveolar lavage fluid; ND, not detected; NF, the normal flora; WS staining, Warthin-Starry silver staining; +, positive; -, negative. ^#^, Ratio of *T. denticola* reads in *Treponema*. * means the normal flora from the upper respiratory tract in BALF sample. The interpretation of pathogens and normal flora are based on references from The ABX Guide and Manual of Clinical Microbiology (11th Edition).

### 3.4 Histopathological validation by Warthin-Starry staining

To obtain direct morphological evidence of spirochete presence, we performed Warthin-Starry silver staining on archived formalin-fixed, paraffin-embedded lung tissue samples from four patients who underwent surgical resection or biopsy (Cases 1, 2, 4, and 5). Notably, three of these four cases (Cases 1, 4, and 5) demonstrated positive staining, revealing abundant black, spiral-shaped bacteria within the necrotic cores of the pulmonary abscesses (**Figure 2**).

### 3.5 Treatment and prognosis

One patient achieved near-complete radiographic resolution after a 2-month course of amoxicillin-clavulanate, while another case showed significant improvement with residual fibrous streaks following 7 months of doxycycline therapy. The remaining 5 patients were initially treated with quinolone drugs (levofloxacin or moxifloxacin), with divergent clinical outcomes. Among them, 3 cases were converted to surgical resection of the lesion due to poor response to medical therapy, including one case complicated by massive hemoptysis. All surgical specimens exhibited histopathological features of chronic lung abscess, including dense neutrophilic and lymphocytic infiltration with micro-abscess formation, alveolar histiocyte accumulation, and organizing pneumonia (**Figure 2**). These patients achieved complete recovery post-resection. One patient developed concomitant nontuberculous mycobacterial (NTM) infection during moxifloxacin treatment, necessitating conversion to a regimen of rifampin, ethambutol, and azithromycin, though with persistent infection. Another case showed marked improvement after switching from ineffective moxifloxacin to a 6-month sitafloxacin course, ultimately achieving infection control with residual cavitary changes (**Figure 1**).

### 3.6 Literature review

Systematic literature retrieval identified one documented case of *T. denticola*-associated lung abscess in each of PubMed and CNKI databases, with both cases presenting poor periodontal health and active dental disease (Guo et al., 2019; Bao and Tang, 2021). Hemoptysis was the predominant symptom in both cases. Chest CT imaging revealed typical lung abscess formation. mNGS analysis of biopsy tissues suggests *T. denticola* is very possible as one of the potential pathogens. The initial treatment of one case was moxifloxacin, and there was no significant clinical improvement. Following diagnosis, intravenous penicillin G was administered for a 6-week (Bao and Tang, 2021). Follow-up imaging revealed marked abscess resolution. The other patient received a 2-month course of metronidazole plus oral amoxicillin/clavulanate, with subsequent significant reduction in pulmonary mass size post-treatment (Guo et al., 2019).

## 4 Discussion

Reports of *Treponema denticola*-associated lung abscess are exceedingly rare, with only two documented cases worldwide to date (Guo et al., 2019; Bao and Tang, 2021). Clinicians’ understanding of this condition remains highly limited. This study presents seven cases of *Treponema denticola* lung abscess confirmed by metagenomic next-generation sequencing (mNGS), systematically analyzing their clinical manifestations, diagnostic approaches, and treatment outcomes. These findings provide crucial insights for the diagnosis and management of this rare infection.

It is reported that patients with poor oral health (periodontal disease) and compromised immune systems are more likely to develop lung abscesses (Guo et al., 2019; Takayanagi et al., 2010), with over 60% of lung abscess patients found to have periodontal disease. As a common anaerobic bacterium in the oral cavity, *T. denticola* may translocate to the lungs through micro-aspiration, potentially establishing infection in the setting of impaired host defenses (Zhang et al., 2024; Shao et al., 2023). In this series of seven cases, none of the patients had significant immunosuppression, but all exhibited poor oral hygiene with significant dental plaque and calculus accumulation, which can lead to dysbiosis of the oropharyngeal microbiota and increase the risk of colonization and proliferation of opportunistic pathogens. Moreover, our patients were predominantly elderly (with an average age of 62.3 years), as age-related declines in both cough reflex sensitivity and immune competence create favorable conditions for opportunistic pulmonary infections. Interestingly, although alcohol abuse has been established as a recognized risk factor for lung abscess (Vaarst et al., 2023; Mohapatra et al., 2018), it did not emerge as a significant contributor in our series, with only one male patient reporting substantial alcohol consumption.

The clinical presentation of *T. denticola-*associated lung abscesses demonstrates several distinctive features. Patients typically exhibit non-specific respiratory symptoms, with cough and sputum production being predominant manifestations, while systemic inflammatory responses remain relatively mild. The disease course tends to be indolent, as evidenced by our cohort’s mean diagnostic delay of 3.6 months. Such mild inflammatory parameters (normal or slightly elevated biomarkers) frequently lead to misdiagnosis as non-infectious conditions (e.g., tuberculosis or lung cancer), likely reflecting the low virulence and chronic infection patterns characteristic of *T. denticola*.

Radiologically, chest CT mainly presents as pulmonary mass accompanied by liquefactive necrosis and small cavity formation, typically without air-fluid levels - a potentially distinguishing feature from conventional pyogenic abscesses. A striking clinical observation was the high prevalence of hemoptysis (85.7%, 6/7 cases), including one case of life-threatening massive hemoptysis. This incidence markedly exceeds the 21.7% hemoptysis rate reported in typical lung abscesses (Montméat et al., 2024), a finding corroborated by the two previously reported cases where hemoptysis was also the cardinal symptom (Guo et al., 2019; Bao and Tang, 2021).

The development of tissue necrosis and hemoptysis in *T. denticola* infections likely results from a complex interplay of bacterial virulence factors and host inflammatory responses. Key virulence determinants, including major surface protein (MSP) and chymotrypsin-like protease complex (CTLP), directly compromise cellular integrity through degradation of extracellular matrix (ECM) components, leading to substantial tissue damage (Dashper et al., 2011). Additionally, CTLP, MSP, lipooligosaccharides, and the cysteine-desulfhydrase enzymes HlyA (also known as Cystalysin) contribute to the pathogenic process by exhibiting cytotoxic effects that promote cellular dysfunction and death (Chu et al., 1999). Concurrently, *T. denticola* infection elicits a robust local inflammatory response characterized by macrophage activation and elevated secretion of proinflammatory mediators, particularly IL-6, IL-8, and RANTES. This inflammatory cascade is further amplified through activation of multiple intracellular signaling pathways, including PKA, ERK2, JNK, and p38, which collectively enhance production of inflammatory cytokines (Kimizuka et al., 2003; Asai et al., 2003; Deng et al., 2001; Nixon et al., 2000). The combined effects of direct bacterial cytotoxicity and sustained inflammatory stimulation result in significant microvascular injury, manifested by increased vascular permeability, capillary damage, and ultimately vascular rupture.

As an anaerobic pathogen, *T. denticola* has posed significant diagnostic challenges due to its fastidious growth requirements and the technical difficulties associated with anaerobic specimen collection and transport (Fenno, 2012). Conventional culture methods frequently yield false-negative results, particularly when analyzing sputum or upper respiratory secretions that are often contaminated with oral commensal flora (Gao et al., 2025). These limitations have contributed to the underrecognition of anaerobic organisms in pulmonary abscess etiology. Comparing to traditional techniques, mNGS provides an unbiased detection of all microbial nucleic acids in clinical samples, encompassing bacteria, viruses, fungi, and parasites, which proves particularly valuable for identifying anaerobic bacteria, rare organisms, and polymicrobial infections (Li et al., 2018; Shi et al., 2023; Lee et al., 2025). The detection rate of anaerobic bacteria in traditional sputum culture is only 20% -30%, while the detection rate of anaerobic bacteria in mNGS can reach over 80% (Huang et al., 2020; Chen et al., 2021a) While mNGS represents a transformative diagnostic tool for rare pathogens, its clinical application requires careful interpretation (Zhang et al., 2024). Our histopathological findings provide tangible morphological support for the mNGS results. The detection of spiral-shaped bacteria via Warthin-Starry staining in three of four tested cases offers independent visual confirmation of spirochetes within the abscess material (**Figure 2**). Although this special stain cannot specifically identify *T. denticola* at the species level, the combination of positive staining with the species-specific mNGS data creates a compelling chain of evidence. This multi-methodological approach significantly strengthens the etiological linkage between *T. denticola* and the observed pulmonary pathology.

The interpretation of mNGS results in the context of polymicrobial infections warrants careful consideration. In our series, *T. denticola* was detected as the predominant organism in three cases (Cases 3, 5& 7), suggesting a primary pathogenic role. However, in the majority of cases, it was co-detected with other recognized oral pathogens, such as *P. gingivalis* and *S. constellatus.* In these instances, the relative abundance of *T. denticola* varied and was sometimes lower than that of its partners. This finding aligns with the established model of polymicrobial synergy in periodontal disease, particularly within the ‘red complex’ (Mahendrarajan et al., 2025). Within this complex, *T. denticola*, though not always the most abundant, may facilitate infection through its potent virulence factors, such as CTLP, which disrupts host tissues and enables co-colonization by other pathogens (Mahendrarajan et al., 2025; Kamarajan et al., 2024). Therefore, the clinical significance of *T. denticola* likely extends beyond its relative abundance in sequencing data, encompassing its key role in initiating and sustaining a dysbiotic, pathogenic consortium.

*T. denticola* demonstrates susceptibility to multiple classes of antimicrobial agents. *In vitro* studies by Pawar et al. revealed that various *Treponema* species maintain sensitivity to penicillin, tetracycline, imipenem, cefoperazone, clindamycin, and moxifloxacin, while exhibiting partial resistance to metronidazole (Pawar et al., 2024). Kazuko et al. demonstrated that doxycycline, minocycline, azithromycin, and erythromycin exhibit consistent activity against *T. denticola*, *T. socranskii*, and *T. vincentii*. Notably, fluoroquinolones showed limited efficacy, demonstrating activity only against *T. socranskii* while remaining ineffective against both *T. denticola* and *T. vincentii* (Okamoto-Shibayama et al., 2017). This laboratory findings align with our clinical observations. Patients treated with amoxicillin-clavulanate or doxycycline regimens demonstrated favorable therapeutic responses. Conversely, fluoroquinolones (moxifloxacin or levofloxacin) appear to have limited clinical utility against this pathogen. Notably, one representative case showed disease progression during moxifloxacin treatment but subsequently responded well to sitafloxacin (STFX). This observation aligns with the antimicrobial susceptibility findings reported by Kazuko et al., who documented STFX’s superior anti-treponema activity, demonstrating MIC values 4–8 times lower than those of ofloxacin and levofloxacin (Okamoto-Shibayama et al., 2017). The development of quinolone resistance in *T. denticola* appears mediated through multiple molecular mechanisms, including genetic mutations in DNA gyrase and topoisomerase IV, coupled with ATP-binding cassette (ABC) transporter-mediated efflux systems (Okamoto-Shibayama et al., 2017; Hooper and Jacoby, 2016). These findings suggest that while β-lactam/tetracycline-based regimens remain first-line options, the potential role of STFX in quinolone-resistant cases warrants further investigation. Therefore, we should search for the pathogen as early as possible for a clear diagnosis, choose antibiotic treatment that is sensitive to the identified pathogen, and achieve the transition from empirical treatment to targeted treatment. In addition, the results of this study showed that *T. denticola* are prone to co infection with other anaerobic bacteria, enhancing their resistance to antibiotics and host immunity, resulting in poor anti infection effects. Pulmonary abscess caused by *T. denticola* often presents as a chronic disease course with a relatively long overall treatment time. For patients with ineffective internal medicine treatment or massive hemoptysis, surgical resection of the lesion is an effective means of controlling bleeding and clearing the source of infection. In this group of 3 patients (42.9%), the infected lesions were ultimately completely removed through surgical intervention.

Several limitations should be considered about our findings. Firstly, the small sample size (n=7) may introduce selection bias and limit the generalizability of our observations. Furthermore, *T. denticola* was co-detected with other oral commensal bacteria in some cases. However, the limited number of cases precluded further investigation into how such co-infections influence clinical presentation or therapeutic outcomes. A key methodological limitation was the absence of anaerobic culture. Although this reflects real-world clinical challenges, it means we could not obtain cultured isolates for phenotypic confirmation. However, this limitation was mitigated by the histopathological evidence from Warthin-Starry staining, which confirmed the physical presence of spirochetes within the lesions in the majority of tested cases, thereby providing independent validation that aligns with the mNGS results.

## 5 Conclusion

This study represents the first case series analysis of *T. denticola*-associated lung abscesses, providing valuable insights into the diagnosis and management of this uncommon clinical entity. Our findings demonstrate that *T. denticola* lung abscess typically affects individuals with poor oral health, presenting as indolent infections with prominent hemoptysis. Typical CT findings include a mass-like lesion with cavitation but no air–fluid level. Traditional microbiological methods frequently fail detection, establishing mNGS as the diagnostic cornerstone. The antibiotics including β-lactams and tetracyclines represent optimal antimicrobial choices, while fluquinolones demonstrate restricted utility. Surgical management remains crucial for refractory cases or life-threatening hemoptysis. Though providing valuable insights into this uncommon infection, our findings warrant corroboration through larger-scale investigations.

## Data Availability

The datasets presented in this study can be found in online repositories. The names of the repository/repositories and accession number(s) can be found below: https://www.ncbi.nlm.nih.gov/, PRJNA1305147.
