# Supplementary material for: Clinical characteristics of Treponema denticola-associated lung abscess diagnosed by metagenomic next-generation sequencing: a case series analysis
Source: Front Cell Infect Microbiol. 2025 Nov 11;15:1688498. doi: 10.3389/fcimb.2025.1688498 (PMC12644101; doi:10.3389/fcimb.2025.1688498)
Supplement: Supplementary file 1 [file Table1.docx]

**Supplementary Table 1. Oral status assessment of patients with *Treponema denticola* lung abscesses.**

| Case | Periodontal Disease | Decayed Tooth | Plaque Index* | Simplified Calculus Index* | Poor oral hygiene |
| --- | --- | --- | --- | --- | --- |
| 1 | N | N | 4 | 2 | Dental calculus*, dental plaque# |
| 2 | N | N | 4 | 2 | Dental calculus*, dental plaque# |
| 3 | N | Y | 3 | 1 | Decayed tooth |
| 4 | N | N | 3 | 2 | Dental calculus*,dental plaque# |
| 5 | N | Y | 3 | 1 | Decayed tooth |
| 6 | Y | N | 4 | 2 | Periodontal disease, dental calculus* |
| 7 | Y | N | 4 | 2 | Periodontal disease, dental calculus* |
| Mean and SD | / | / | 3.57 ±0.53 | 1.71±0.49 |  |

N, no; Y, yes；SD, standard deviation. *, Dental calculus and plaque were evaluated according to the Chinese Stomatological Association's guideline. Plaque Index was scored on a 0-5 scale: 0, no plaque; 1, scattered plaque at the gingival margin; 2, a continuous narrow band of plaque (≤1 mm) at the gingival margin; 3, plaque covering >1 mm but <1/3 of the tooth surface; 4, plaque covering ≥1/3 but <2/3 of the tooth surface; 5, plaque covering ≥2/3 of the tooth surface. Simplified Calculus Index was scored on a 0-3 scale: 0, no calculus; 1, supragingival calculus covering <1/3 of the tooth surface; 2, supragingival calculus covering between 1/3 and 2/3 of the tooth surface, or sporadic subgingival calculus; 3, supragingival calculus covering ≥2/3 of the tooth surface, or a continuous heavy band of subgingival calculus.
