# Supplementary material for: Clinical characteristics of Treponema denticola-associated lung abscess diagnosed by metagenomic next-generation sequencing: a case series analysis
Source: Front Cell Infect Microbiol. 2025 Nov 11;15:1688498. doi: 10.3389/fcimb.2025.1688498 (PMC12644101; doi:10.3389/fcimb.2025.1688498)
Supplement: Supplementary file 2 [file Table2.docx]

**Supplementary Table 2. Species-level composition of the *Treponema* genus identified by mNGS in each patient sample.**

| Case No. | Total *Treponema*   Reads | *Treponema*  Species | Species-Specific Reads | Relative Abundance |
| --- | --- | --- | --- | --- |
| **1** | 45,309 | *T. denticola* | 10,077 | 22.2% |
|  |  | *Other *Treponema* spp. | 35232 | 77.8% |
| **2** | 91 | *T. denticola* | 39 | 42.9% |
|  |  | *T. lecithinolyticum* | 13 | 14.3% |
|  |  | *Other *Treponema* spp. | 39 | 42.9% |
| **3** | 10,433 | *T. denticola* | 5428 | 52.0% |
|  |  | *T. lecithinolyticum* | 1724 | 16.5% |
|  |  | *Other *Treponema* spp. | 3281 | 31.4% |
| **4** | 555,456 | *T. denticola* | 145841 | 26.3% |
|  |  | *T. socranskii* | 138600 | 25.0% |
|  |  | *Other *Treponema* spp. | 271015 | 48.7% |
| **5** | 6,105,122 | *T. denticola* | 3718018 | 60.9% |
|  |  | *T. lecithinolyticum* | 982168 | 16.1% |
|  |  | *Other *Treponema* spp. | 1404936 | 23.0% |
| **6** | 139,200 | *T. denticola* | 62361 | 44.8% |
|  |  | *Other *Treponema* spp. | 76775 | 55.2% |
| **7** | 182,982 | *T. denticola* | 86001 | 47.0% |
|  |  | *Other *Treponema* spp. | 96981 | 53.0% |

mNGS, metagenomic next-generation sequencing. “*Other *Treponema* spp.” includes all reads mapped to the *Treponema* genus that could not be classified to a specific species.
